# Supplementary material for: The TOX2 Gene Is Responsible for Conidiation and Full Virulence in Fusarium pseudograminearum
Source: Curr Issues Mol Biol. 2025 Sep 2;47(9):714. doi: 10.3390/cimb47090714 (PMC12468165; doi:10.3390/cimb47090714)
Supplement: Supplementary file 1 [file cimb-47-00714-s001.zip › cimb-3819064-supplementary.pdf]

Supplementary Information

The TOX2 Gene is responsible for conidiation and full virulence in *Fusarium pseudograminearum*

Sen Han[1], Shaobo Zhao[2], Yajiao Wang[1], Qiusheng Li[1], Mengwei Sun[1], Lingxiao Kong[1], Xianghong Chen[1], Jianhai Gao[3], Yuxing Wu[1*]

[1]Plant Protection Institute, Hebei Academy of Agriculture and Forestry Sciences/ Key Laboratory of Integrated Pest Management on Crops in Northern Region of North China, Ministry of Agriculture and Rural Affairs, P. R. China/ IPM Innovation Center of Hebei Province/ International Science and Technology Joint Research Center on IPM of Hebei Province, Baoding, China; hansen19920603@163.com(S.H.); yajiaowang515@163.com(Y.W.); alidd@163.com(Q.L.); 17330283350@163.com(M.S.); konglingxiao163@163.com(L.K.); 13931226235@163.com(X.C.)

[2]Plant Protection and Quarantine General Station of HeBei province China; zhaoshaobo@163.com(S.Z.)

[3]Bureau of Agriculture and Rural of Cangxian, Cangzhou, China; cxnyjyqb@163.com(J.G.)

*Correspondence: wyx1209@163.com

Table S1. Primers used in this study

Primer	Sequence[a] (5'-3')	Use
FpTox2 1F	ACAGTAAGCTGTCCGTTA	To amplify upstream homologue arm of *FpTox2*
FpTox2 2R	TTGACCTCCACTAGCTCCAGCCAAGCCAGATTTGCAAATAGGATAGA	
FpTox2 3F	GAATAGAGTAGATGCCGACCGCGGGTTTCTGTGCCTTGGTTTATGAT	To amplify downstream homologue arm of *FpTox2*
FpTox2 4R	AGATACTTATACCTATCTGT	
HYG F	GGCTTGGCTGGAGCTAGTGGAGGTCAA	To amplify *HYG* gene
HYG R	AACCCGCGGTCGGCATCTACTCTATTC	
FpTox2 5F	TCTTGTCACCATGACAT	To amplify a 313-bp fragment of *FpTox2*
FpTox2 6R	AAAGTTGACTGTCAACT	
H850	TTGTCCGTCAGGACATTGTT	To amplify a 598-bp upstream homologue arm of *HYG*
H852	AACTCACCGCGACGTCTGTC	
FpTox2 7F	AATTATTCAGCATCAGAT	To amplify a 1353-bp fragment
H855R	GCTGATCTGACCAGTTGC	
H856F	GTCGATGCGACGCAATCGT	To amplify a 1186-bp fragment
FpTox2 8R	ACAGTTAATGCCTAAACCT	
FpTox2 9F	TCTCATCACCATCACCATCACAACCTTATAAAAACGTAGT	To amplify a fragment of *FpTox2* and its promoter region
FpTox2 10R	TCGCCCTTGCTCACCCTCGATCACTTATCGTCGTCATCCTTGTAATCACATGCTCCCTGGCAACT	
FpTox2 RTF	CGGCTCAGGACTATGCTCTG	Real-time PCR
FpTox2 RTF	ACATGCTCCCTGGCAACTAG	
FpTEF1-RTF	TCACCACTGAAGTCAAGTCC	Real-time PCR
FpTEF1-RTR	ACCAGCGACGTTACCACGTC	

Table S2. Sequence of FpTox2

DNA sequence
ATGCATTACTCTACTCTTACTACCCTTTCCGCTCTTGTCACCATGACATCCGCTCTCGG<br>AATCAACTGTCGCGGCTCAGGACTATGCTCTGGTGGCGCCGGCAACCTCATCAATCT<br>GAAAGCAATTGTCGACAACATTCAACCACGAGACCGCCACTATGAGACAGGCCAGC<br>AAGTCGCTTGTACTGGTGACACTTGCGCTTTCTTTCAGAGTAGCGCAACCGGTACTG<br>CTGATGATGTGTCCTGGGCCCTACAGGCCCTTCTTGACCACGGCTGTAAGAAGTGTG<br>GGTCTGTCCCCATGCAGGATGGCAACAACGTCGATGATGGGCAGTTGACAGTCAAC<br>TTTGTCAGCGACCCTAGTTGCCAGGGAGCATGTTAA
Protein sequence
MHYSTLTTLSALVTMTSALGINCRGSGLCSGGAGNLINLKAIVDNIQPRDRHYETGQQVACT<br>GDTCAFFQSSATGTADDVSWALQALLDHGCKKCGSVPMQDGNNVDDGQLTVNFVSDPSCQ<br>GAC
